# Distribution, course, and spatial relationships of the saphenous nerve: A 3D neuroanatomical map for nerve stimulation

**DOI:** 10.1371/journal.pone.0297680

**Published:** 2024-02-08

**Authors:** Michael Peng, Paul B. Yoo, Anne M. R. Agur

**Affiliations:** 1 Department of Surgery, University of Toronto, Toronto, Ontario, Canada; 2 Institute of Biomaterials and Biomedical Engineering, University of Toronto, Toronto, Ontario, Canada; 3 Department of Electrical and Computer Engineering, University of Toronto, Toronto, Ontario, Canada; Szegedi Tudomanyegyetem, HUNGARY

## Abstract

The overall objective of this study was to construct a 3D neuroanatomical map of the saphenous nerve based on cartesian coordinate data to define its course in 3D space relative to bony and soft tissue landmarks. Ten lower limb embalmed specimens were meticulously dissected, digitized, laser scanned, and modelled in 3D. The course of the main branches, number of collateral branches, and relationship of saphenous nerve to the great saphenous vein were defined and quantified using the high-fidelity 3D models. In 60% of specimens, the saphenous nerve was found to have three branches in the leg, infrapatellar, anterior, and posterior. In 40% of specimens, the posterior branch was absent. Three landmarks were found to consistently localize the anterior branch: the medial border of tibia at the level of the tibial tuberosity, the medial border of tibia at the level of the mid-point of leg, and the mid-point of the anterior border of the medial malleolus. The posterior branch, when present, had variable branching patterns but did not extend as far distally as the medial malleolus in any specimen. Anatomically, the anterior and posterior branches at the level of the tibial tuberosity could be most advantageous for nerve stimulation due to their close proximity to the bifurcation of the saphenous nerve where the branches are larger and more readily localizable than distally. Additionally, the tibial tuberosity is a prominent landmark that can be easily identified in most individuals and could be used to localize the anterior and posterior branch using ultrasound or other imaging modalities. These findings will enable implementation of highly realistic computational models that can be used to simulate saphenous nerve stimulation using percutaneous and implanted devices.

## Introduction

Overactive bladder (OAB) is a chronic condition that is characterized by symptoms of urgency, frequency, nocturia and urinary incontinence [1]. The prevalence of OAB increases with age and affects up to 16.5% of the US population, with a similar prevalence in females (16.9%) and males (16.0%) [2]. OAB negatively impacts the patient’s daily and recreational living, mental health, and quality of sleep [3]. The total economic burden in the United States is estimated at $65.9 billion, where $14.6 billion are indirect costs due to lost productivity [4].

Saphenous nerve (SN) stimulation is a novel therapeutic approach aimed at treating OAB patients as an alternative to pharmacologic treatment [5]. The SN is a peripheral nerve which carries somatic sensory, visceral afferent, and sympathetic motor nerve fibres. Currently, only one pilot feasibility study of percutaneous SN stimulation has been conducted on a group of 16 patients with OAB [5]. In this study, SN was targeted 1.5 in below and anterior to the medial tibial condyle. An 87.5% positive response rate was reported, based on at least a 50% reduction in bladder symptoms or a 10 point increase in the health related quality of life (HRQL) part of the Overactive Bladder Questionnaire (OAB-q), with no adverse effects.

Further investigation of this technique is needed to determine its anatomical efficacy to inform future clinical studies. Four cadaveric studies were found that investigated the course and branching of SN in the leg [6–9], however, only two studies measured the distance of SN from anatomical landmarks [6, 7]. These studies primarily investigated the main branches and a select number of collateral branches, with limited reference to anatomical landmarks that could be used to localize SN and its branches.

No studies have mapped the course and branching of SN volumetrically in 3D. Simplified models of the leg limit the clinical translatability to design and engineer innovative nerve stimulation methods, such as implantable or non-invasive devices for the treatment of OAB. More sophisticated models require precise 3D digitized data of nerves and their branches, blood vessels, and other soft tissue elements *in situ* in the human lower limb. Current anatomical data are not adequate for the construction of high-fidelity finite element models to optimize electrode placement protocols for SN stimulation.

Therefore, the overall objective of this study was to construct a 3D neuroanatomical map of SN to define its course in 3D space relative to bony and soft tissue landmarks. The results will enable us to implement realistic computational models that can subsequently be used to simulate various forms of electrical stimulation of the human SN.

## Materials and methods

Ten lower limb embalmed cadaveric specimens (5M/5F) with a mean age of 79.2 ± 9.5 years were used in this study. The specimens had no visible signs of pathology, edema, or previous surgery. Approval for this study was obtained from the University of Toronto Health Sciences Research Ethics Board #27210. The methodology to collect 3D data of SN in the lower limb consisted of dissection, digitization, and laser scanning, followed by 3D modelling and data analysis. The Microscribe® G2X Digitizer (Immersion Corporation, San Jose, CA) was used to collect cartesian coordinate data of SN and related structures (accuracy of 0.23mm). The neurovascular structures were digitized at 1-2cm increments and the bony and soft tissue landmarks in a grid pattern. Specimens were laser scanned with the Faro® Laser ScanArm® (FARO Technologies Inc, Lake Mary, FL) throughout the dissection and digitization process to enable reconstruction of 3D meshes from point cloud data.

### Dissection, digitization, and laser scanning protocol

The skin was removed from the entire lower limb, leaving the subcutaneous tissue intact. The knee joint was stabilized in neutral with metal strapping. Three screws were drilled into bony landmarks to serve as reference markers for calibration and later reconstruction of the digitized data. Next, the great saphenous vein (GSV) was localized proximally at the saphenous opening. The GSV was exposed throughout its course in the subcutaneous tissue of the thigh and leg to the dorsal venous arch of the foot and then digitized in its entirety.

After digitization of the GSV, the SN was localized in the subcutaneous tissue at the knee joint line medially. The SN and each of its branches were traced in the subcutaneous tissue from the knee joint line to the foot and digitized throughout their entire course. Next, the subcutaneous tissue was removed to expose the deep fascia of the thigh (fascia lata) and leg (crural fascia). Following exposure of the deep fascia, the lower limb was laser scanned. The anterior border and medial surface of the tibia, patella, and patellar ligament were delineated and their surfaces digitized in a grid pattern.

Following dissection and digitization of SN and its branches in the subcutaneous tissue of the leg, the sartorius was reflected to enable tracing of the SN proximally in the subsartorial canal to its origin from the femoral nerve at the inguinal ligament. Next, the SN was digitized from the inguinal ligament to the medial knee joint line. Photographs were taken throughout the dissection process. Black sutures, that were later digitally colourized, were placed under the SN and its branches to enhance visualization.

### 3D modelling of digitized data

The digitized data of SN and its branches, GSV, patella, patellar ligament, medial surface of the tibia of each specimen were imported into Autodesk® Maya® 2019 (Autodesk Inc., San Rafael, CA). Nerve branches and vessels were modelled into tubes using a non-uniform rational B-spline (NURBS) circle extrusion script developed in the laboratory. Bony and connective tissue elements were lofted to create a 3D surface from the grid pattern produced in the digitization process. Laser scan data were reconstructed into 3D surface meshes using MeshLab and then imported into Autodesk® Maya® 2019. These layers were fitted using reference points for each specimen. The model of each specimen consisted of SN and its branches, GSV, the surface of the crural fascia, and bony and connective tissue surfaces of the tibia, patella, and patellar ligament.

### Data analysis

Using 3D models and photographs of the dissected specimens, the main and collateral branches of SN were identified and their course documented. The number of main and collateral branches were quantified and the frequency of each of the identified branching patterns was determined. The area of termination of the main branch(es) and their relationship to the medial malleolus was documented. The relationship of the main branch(es) to the GSV in the proximal, middle, and distal thirds of the leg was determined for each specimen and summarized in a frequency table.

Bony landmarks that could be used to localize the main branches of SN were determined. The distance of the main branch(es) of SN to the bony landmarks was quantified using the measurement tool in Autodesk® Maya® 2019. The following measurements were recorded:

- Distance of SN from medial border of tibia at the level of the tibial tuberosity
- Distance of SN from medial border of tibia at the level of the mid-point of leg
- Distance of SN from mid-point of anterior border of medial malleolus

The branching patterns, the course of the main branch(es) of SN, and their relationship to palpable bony landmarks were used to propose anatomically optimal site(s) to stimulate the SN for treatment of OAB. The proposed sites will be used to inform finite element modelling of SN stimulation.

## Results

**Fig 1:**
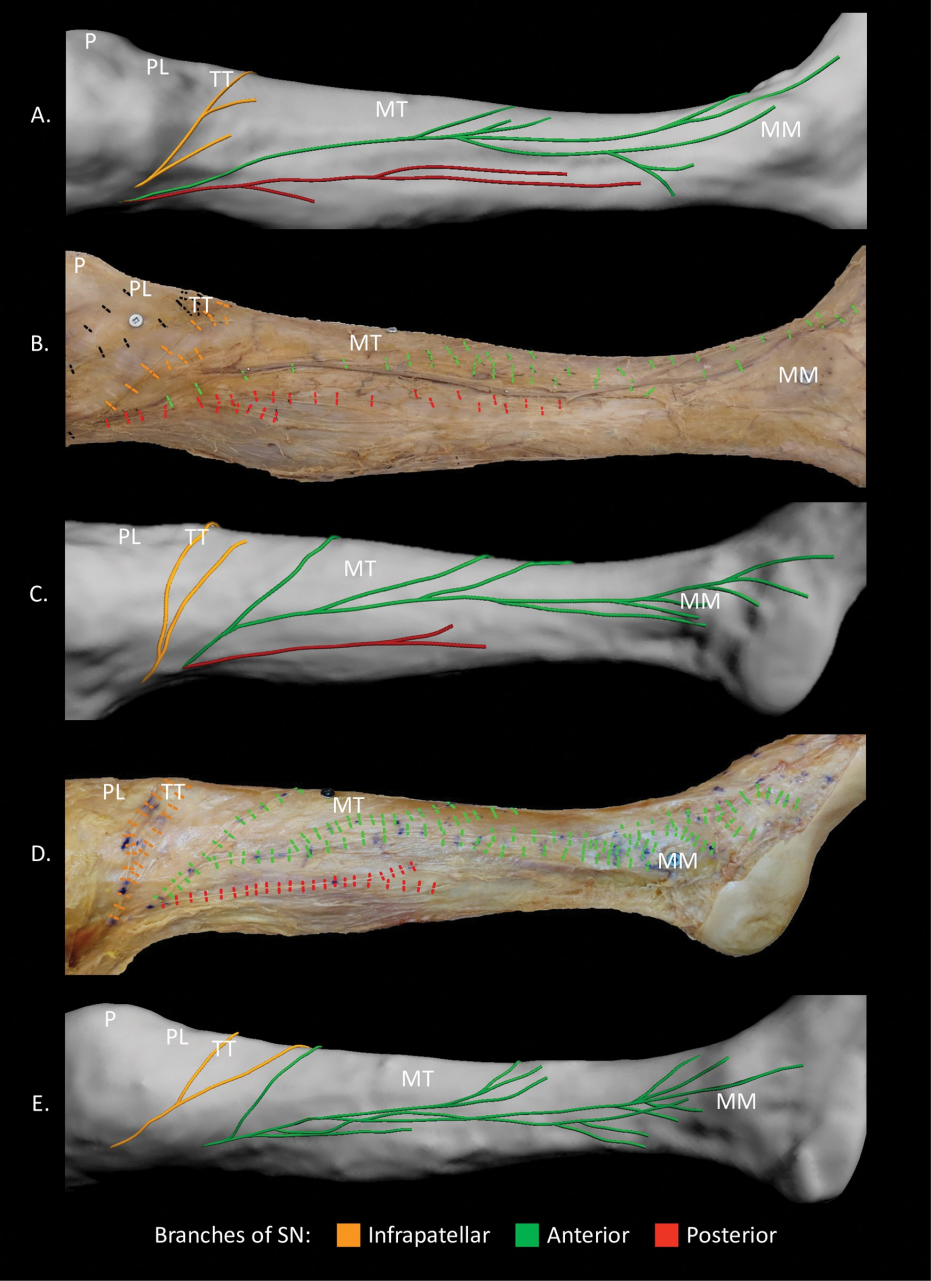
Dissections and 3D models of the branching pattern of the saphenous nerve, medial views. (A) and (B) 3D model and dissection of the same specimen with a long posterior branch. (C) and (D) 3D model and dissection of the same specimen with a short posterior branch. (E) 3D model of specimen with no posterior branch. MM, medial malleolus; MT, medial surface of tibia; P, patella; PL, patellar ligament; TT, tibial tuberosity.

The 3D computer modelling allowed full reconstruction of the main and collateral branches of SN along with the GSV, crural fascia, and bony and soft tissue elements as *in situ*. The nerve branches were displayed throughout the volume of the leg and could be viewed as individual components or in composite to show spatial relationships of nerve branches to bony and soft tissue landmarks (Fig 1).

The SN was found to divide into two or three main branches upon piercing the crural fascia. In the specimens with three main branches (n = 6), the SN divided into an infrapatellar (IP), anterior (AB), and posterior branch (PB) (Fig 1A–1D). In the specimens with two main branches (n = 4), the SN divided into an IP and AB; PB was not present (Fig 1E).

### Infrapatellar branch

**Fig 2:**
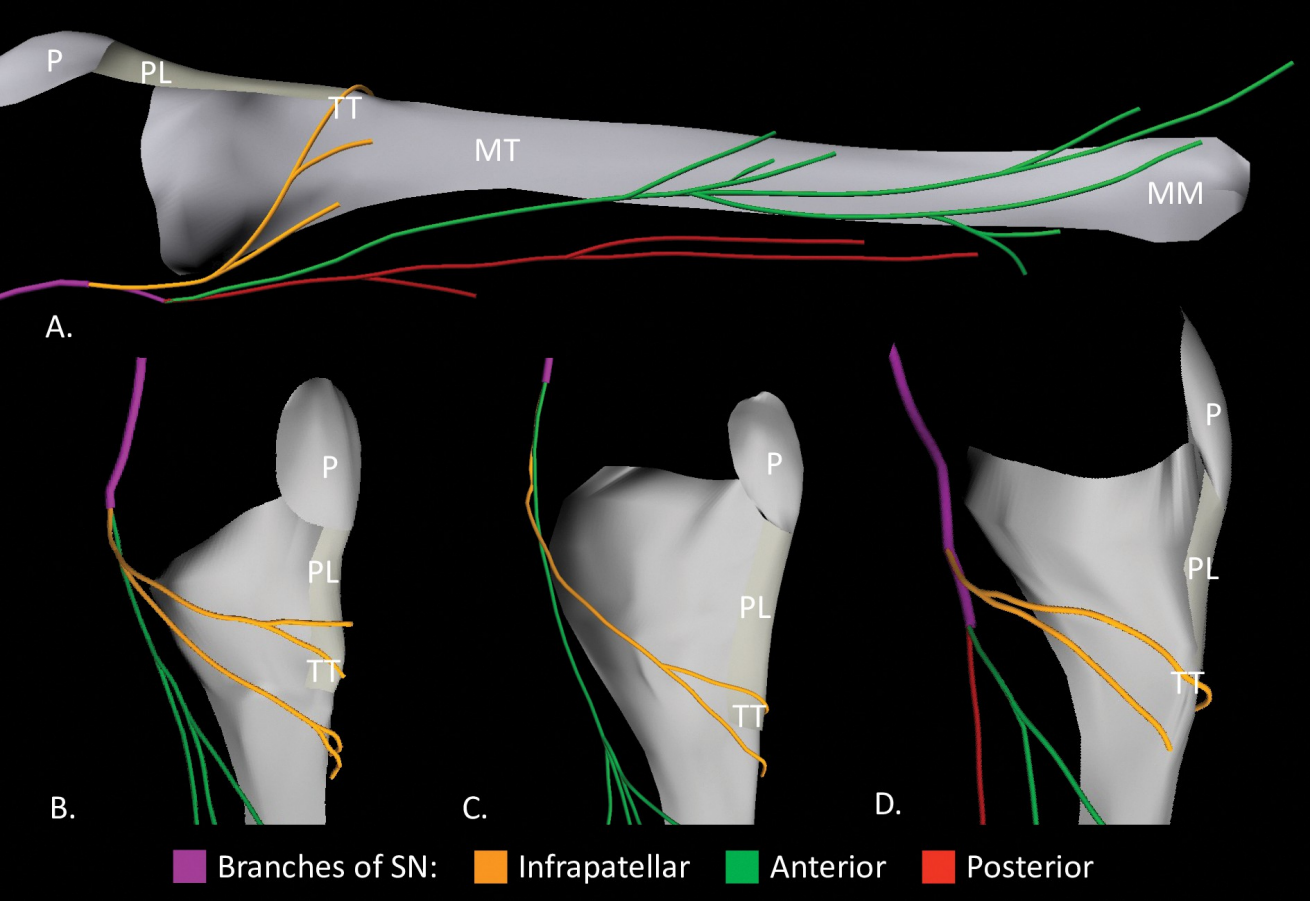
3D models showing the branching of saphenous nerve proximally, medial views. (A) Branching at knee joint line. (B) and (C) Branching proximal to knee joint line. (D) Branching distal to knee joint line. MM, medial malleolus; MT, medial surface of tibia; P, patella; PL, patellar ligament; TT, tibial tuberosity.

The IP was given off the SN superior to the medial knee joint line (n = 6), at the level of the medial knee joint line (n = 2), or inferior to the medial knee joint line (n = 2) (Fig 2). The IP coursed inferolaterally in the thin layer of subcutaneous tissue overlying the medial surface of the tibia. On average, IP had 3.2 ± 1.2 (range: 2–5) collateral branches (S1 Table). The collateral branches coursed:

- superior to the tibial tuberosity (n = 1).
- at the level of the tibial tuberosity (n = 3).
- inferior to the tibial tuberosity (n = 4).
- at the level of and inferior to tibial tuberosity (n = 2).

### Anterior branch

**Fig 3:**
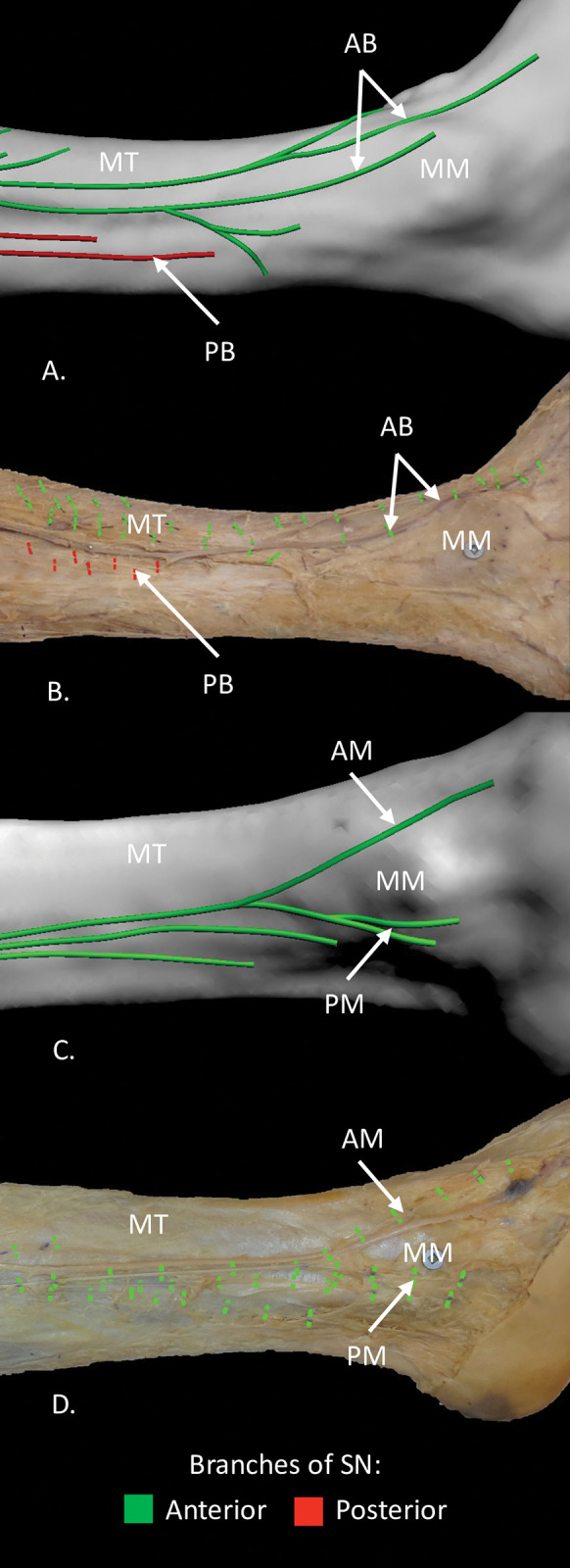
Distributions of distal branches of saphenous nerve at the ankle, medial views. (A) and (B) 3D model and dissection of the same specimen showing the long posterior branch terminating proximal to the medial malleolus and the anterior branch coursing anterior to the medial malleolus. (C) and (D) 3D model and dissection of the same specimen showing the anterior branch dividing to course anterior and posterior to the medial malleolus, no long posterior branch was present. AB, anterior branch of saphenous nerve; AM, anterior malleolar branch; MM, medial malleolus; MT, medial surface of tibia; PB, posterior branch of saphenous nerve; PM, posterior malleolar branch.

The AB was present in all specimens and was given off the SN superior to the medial knee joint line (n = 5), at the level of the medial knee joint line (n = 3), or inferior to the medial knee joint line (n = 2) (Fig 2). The AB coursed inferiorly in the subcutaneous tissue along the medial margin of the tibia, turned anteriorly to course over the medial surface of the tibia, and then continued inferiorly to lie anterior to the medial malleolus (Fig 1). In three specimens, the AB turned anteriorly onto the medial surface of the tibia in the middle third of the leg and in seven specimens in the distal third of the leg. At the medial malleolus, the AB bifurcated into anterior and posterior malleolar branches in six specimens and remained a single branch in four specimens (Fig 3).

The mean number of collateral branches given off AB was 8.3 ± 2.5 (range: 5–13) (S1 Table). Collateral branches originated in the proximal, middle, and distal thirds of the leg in six specimens, the middle and distal thirds in three specimens, or the proximal and distal thirds in one specimen (Fig 1). The collateral branches given off the anterior aspect of the AB coursed anteroinferiorly in the subcutaneous tissue overlying the medial surface of the tibia, with some branches coursing as far as tibialis anterior. Those given off the posterior aspect of AB coursed posteroinferiorly in the subcutaneous tissue along the medial aspect of the gastrocnemius.

### Posterior branch

The PB was found in 60% (6/10) of specimens, originating superior to the medial knee joint line (n = 3) at the level of the medial knee joint line (n = 1), or inferior to the medial knee joint line (n = 2) (Fig 2). The PB coursed inferiorly in the subcutaneous tissue of the posteromedial aspect of the leg to terminate in the proximal half of the leg in four specimens (Fig 1C and 1D) and just superior to the medial malleolus in two specimens (Fig 1A and 1B). The PB gave off 2.2 ± 0.4 (range: 2–3) collateral branches in the proximal third of the leg in three specimens, middle third in two specimens, or in both the proximal and middle thirds in one specimen (S1 Table).

### SN-GSV relationship

In nine specimens, there was a single GSV and in one specimen two GSVs running parallel to each other were present (Fig 1B). In specimens where there was a single GSV, the SN was deep to the GSV separated from it by a thin layer of subcutaneous tissue at the knee joint line. From the level of the tibial tuberosity to the medial malleolus, the AB and GSV lay in close proximity with no intervening subcutaneous tissue. In the proximal third of the leg, the AB was most frequently found anterior or posterior to the GSV, in the middle third of the leg, the AB was primarily posterior or deep, and in the distal third the AB was predominantly deep (Table 1). In specimens with two parallel GSVs, both veins coursed superficial to AB and its branches. The PB, when present, was located posterior to the GSV in all specimens. Proximally, the IP and its branches coursed anterior to the GSV.

**Table 1:** Relative position of SN to GSV in the proximal, middle, and distal thirds of the leg.

Third of leg	Relative position of SN to GSV^a^		
	Posterior	Deep	Anterior
Proximal	3/9 (33%)	2/9 (22%)	4/9 (44%)
Middle	3/9 (33%)	4/9 (44%)	2/9 (22%)
Distal	2/9 (22%)	5/9 (56%)	2/9 (22%)

^a^SN was not found superficial to GSV.

### Localization of AB and PB relative to bony landmarks

The relationship of AB could be quantified relative to bony landmarks including the medial border of tibia at the level of the tibial tuberosity (Fig 2), medial border of the tibia at mid-shaft level (Fig 2A), and mid-point of anterior border of medial malleolus (Fig 3) could be quantified (Table 2). The relationship of the short and long PB could only be quantified relative to the medial border of the tibia at the level of the tibial tuberosity and only that of the long PB to the medial border of the tibia at mid-shaft level (Table 2). The PB did not reach the medial malleolus.

**Table 2:** Relationship and mean distance of AB and PB from bony landmarks.

Landmark	Branches	Relationship to landmark	Distance: Mean ± SD (cm)
Medial border of tibia at the level of the tibial tuberosity	AB, PB	AB: Posterior (n = 10)	AB: 2.02 ± 0.71
		PB: Posterior (n = 6)	PB: 2.80 ± 1.01
Medial border of tibia at the level of the mid-point of leg	AB, PB	AB: Posterior (n = 9)	AB: 1.09 ± 0.53
		PB: Posterior (n = 2)	PB: 2.80 ± 1.41
Mid-point of anterior border of medial malleolus	AB	Anterior (n = 5)	0.94 ± 0.34
		Posterior (n = 5)	1.54 ± 0.43

AB, anterior branch; PB, posterior branch.

At the level of the tibial tuberosity, both the AB and PB coursed posterior to the medial border of the tibia, in the subcutaneous tissue superficial to the crural fascia (Fig 2). The AB on average coursed 0.78 cm closer to the medial border of the tibia than the long/short PB (S2 Table).

At the mid-shaft of the tibia, the AB in nine specimens was found to be located at a mean distance of 1.09 cm posterior to the medial border to the tibia (Fig 2A). In one specimen, the AB coursed just anterior (0.71 cm) to the medial border of the tibia. The long PB in two specimens was found to be located further posteriorly from the medial border of the tibia at the mid-shaft level (mean distance 2.80 cm). The AB on average coursed 1.71 cm closer to the medial border of the tibia at the mid-shaft level than the long PB (S3 Table).

The AB in half the specimens was found to course anterior (0.94 cm) to the medial malleolus (Fig 3). In the other five specimens, the AB travelled on the medial malleolus, 1.54 cm from its anterior border (S4 Table).

## Discussion

### Number of main and collateral branches of SN

The number of main branches of SN were only investigated in four previous studies with variable findings (Table 3). Two studies reported the presence of one main SN nerve [6, 7]. However, similar to the current study, Veverková et al. [8] observed that SN bifurcated into two main branches in the majority of specimens (86.0%) or remained as a single nerve in 14.0% of specimens. Wilmot and Evans [9] found the number of main branches of SN could be categorized into three main types of branching patterns. The three types included: Bifurcation into AB and PB between the knee fold and medial malleolus in 41% of specimens (Type A), bifurcation into AB and PB at the knee fold in 45% (Type B), and bifurcation into a larger-calibre AB and a smaller-calibre PB that coursed posteriorly in 14% (Type C). Similar to Wilmot and Evans [9], the current study found that the AB and PB varied in bifurcation location, length, and calibre. In contrast, in the current study, the PB when present was found to originate superior or at the knee joint line in 66.7% of specimens and inferior to the knee joint line in only 33.3% of specimens.

**Table 3:** Summary of the number of branches of SN.

Author (Year)	Branches of SN		
	Number of main branches^b^	Number of collateral branches	Number of terminal branches at medial malleolus
Wilmot & Evans (2013)	2 (100%)	1 to 5	NR
Veverková et al. (2011)	1 (13.95%) or 2 (86.05%)	NR	NR
Dayan et al. (2008)	1 (100%)	3 to 6	1 (5%) or 2 (95%)
Ghosh & Chaudhury (2019)	1 (100%)	NR	1 (9.52%) or 2 (90.48)
**Current study**	1 (40%) or 2 (60%)	AB: 8.3 ± 2.5	1 (40%) or 2 (60%)
		PB: 2.2 ± 0.4	

AB, anterior branch; PB, posterior branch; NR, not reported.

^b^This table does not include the infrapatellar branch as it was not documented in all studies.

The collateral branches originating from the main branches of SN, AB and/or PB, were previously found and documented using variable criteria, making comparison of results difficult. Veverková et al. [8] stated that “in every case, the SN had a large number of branches with a small calibre that ran in an anterolateral or posterolateral direction in the middle third of the lower leg.” The total number of collateral branches for each of the three branching patterns was documented by Wilmot and Evans [9]. Type A and Type C branching patterns, most commonly, had 2 collateral branches with a range of 1–4 and 1–5 collateral branches respectively. The majority of specimens with Type B branching pattern had one collateral branch (range 1–2). Dayan et al. [6] found three collateral branches in all specimens, the middle-posterior, middle-anterior, and inferior-anterior. The middle-posterior and middle-anterior branches were given off in the superior third of the leg, proximally and distally respectively, and the inferior-anterior branch in the middle third of the leg. Other collateral branches were found in 10–35% of specimens. In contrast, the current study found a greater number of collateral branches from the AB (8.3 ± 2.5) and from the PB (2.2 ± 0.4). Similar to Dayan et al., [6] the collateral branches given off AB were projected in both anterior and posterior directions throughout the leg.

Dayan et al. [6] and Ghosh and Chaudhury [7] found the SN to be a single nerve which divided into anterior and posterior (malleolar) branches in 95% and 90.48% of specimens respectively. In the current study, the AB divided into anterior and posterior malleolar branches in 60% of specimens. In the remaining 40%, the AB continued to pass anterior to the medial malleolus giving off collateral branches that coursed posteriorly over the medial malleolus.

### Landmarks for electrode placement sites for SN stimulation

The dissection and 3D modelling results of the current study suggested three possible viable electrode placement sites, including the medial border of tibia at the level of the tibial tuberosity, medial border of tibia at the level of the mid-point of leg, and mid-point of anterior border of medial malleolus.

The AB and PB could be stimulated just distal to their bifurcation point from the SN at the medial border of the tibia at the level of the tibial tuberosity. Most specimens had not given off collateral branches at this point (n = 7) and in the remaining three specimens only one collateral branch had emerged.

At the medial border of the tibia at the level of the mid-point of leg, the AB and long PB could be stimulated. The AB crosses superficial to the medial border of the tibia and continues to course inferolaterally on the medial surface of the tibia. It is likely that the AB would be stimulated in all specimens and the PB in specimens where it lies in closer proximity to AB.

The anterior malleolar branches of AB could be stimulated at the mid-point of the anterior border of the medial malleolus in all specimens. The posterior malleolar branches may be stimulated if they cross the malleolar surface (n = 4) rather than coursing distinctly posterior to the medial malleolus (n = 2). The short or long PB, when present, terminate proximal to the medial malleolus in the leg.

When considering all three landmarks, stimulation of the AB and PB at the level of the tibial tuberosity could be more optimal than at the other sites. The advantages of this site include:

- The AB and PB are close to their bifurcation from the SN and thus are larger and more readily localizable than more distally, since most collateral branches would have been given off.
- The tibial tuberosity is a prominent landmark from which a horizontal line can be easily extended to the medial border of the tibia.
- The SN, AB, and PB would be most readily identifiable using ultrasound and other imaging modalities at this site due to their size and proximity to prominent landmarks.

Further finite element modelling and *in vivo* studies are needed to develop evidence-based stimulation protocols based on these anatomical findings.

One of the limitations of this study is the small sample size (n = 10). This is due to the time-consuming nature of digitization and 3D modelling. The current study is a foundational study as it can be used to inform finite element modelling and to develop imaging protocols e.g. ultrasound to localize the SN and its branches relative to anatomical landmarks. In future anatomical and *in vivo* studies, the sample size will be increased enabling capture of other possible variations and to determine if there are sex differences. The current study was carried out entirely *in situ* using serial dissection to prevent nerve displacement. Embalmed tissues, for example muscle fibre bundle architecture, as long as dissected *in situ* remains morphologically consistent [10].

## Conclusions

In this novel 3D study, the course and distribution of SN and its branches relative to bony and soft tissue landmarks were modelled, as *in situ*, using cartesian coordinate data obtained from dissection, digitization, and laser scanning of cadaveric specimens. The SN, in 60% of specimens, was found to have three branches in the leg, IP, AB, and PB. In 40% of specimens, the PB was absent. The AB was consistently localizable at: the medial border of tibia at the level of the tibial tuberosity, the medial border of tibia at the level of the mid-point of leg, and the mid-point of anterior border of medial malleolus. The PB when present had variable branching patterns but did not extend as far distally as the medial malleolus in any specimen. Based on the results of this anatomical study, the AB and PB at the level of the tibial tuberosity could be most advantageous for nerve stimulation. Further *in vivo* investigation and finite element modelling is required to determine efficacy of possible electrode placement sites.

## Supporting information

S1 Table Number of collateral branches given off infrapatellar (IP), anterior (AB) and posterior (PB) branches by specimen.
(PDF)

S2 Table Distance of anterior (AB) and posterior (PB) branches from medial border of tibia at level of tibial tuberosity by specimen. (PDF)

S3 Table Distance of anterior branch (AB) from medial border of tibia at level of mid-point of leg by specimen. (PDF)

S4 Table Distance of anterior branch (AB) from mid-point of anterior border of medial malleolus by specimen. (PDF)
