# Supplementary material for: Distribution, course, and spatial relationships of the saphenous nerve: A 3D neuroanatomical map for nerve stimulation
Source: PLoS One. 2024 Feb 8;19(2):e0297680. doi: 10.1371/journal.pone.0297680 (PMC10852217; doi:10.1371/journal.pone.0297680)
Supplement: S1 Table — Number of collateral branches given off infrapatellar (IP), anterior (AB) and posterior (PB) branches by specimen. (PDF) [file pone.0297680.s001.pdf]

S1 Table. Number of collateral branches given off infrapatellar (IP), anterior (AB) and posterior (PB) branches by specimen.

Specimen	IP: # collateral branches	AB: # collateral branches	PB: # collateral branches
**1**	3	8	3
**2**	5	7	PB not present
**3**	2	7	PB not present
**4**	2	13	PB not present
**5**	5	11	2
**6**	2	10	2
**7**	3	5	2
**8**	4	5	2
**9**	2	9	2
**10**	4	8	PB not present
**Mean ± SD**	3.2 ± 1.2	8.3 ± 2.5	2.2 ± 0.4
