# Supplementary material for: Distribution, course, and spatial relationships of the saphenous nerve: A 3D neuroanatomical map for nerve stimulation
Source: PLoS One. 2024 Feb 8;19(2):e0297680. doi: 10.1371/journal.pone.0297680 (PMC10852217; doi:10.1371/journal.pone.0297680)
Supplement: S2 Table — (PDF) [file pone.0297680.s002.pdf]

S2 Table. Distance of anterior (AB) and posterior (PB) branches from medial border of tibia at level of tibial tuberosity by specimen.

Specimen	Distance AB (cm)*	Distance PB (cm)*
**1**	1.7	2.7
**2**	2.2	PB not present
**3**	1.6	PB not present
**4**	2.9	PB not present
**5**	2.2	4.6
**6**	0.4	1.5
**7**	2.2	2.6
**8**	2.5	2.5
**9**	2.7	2.9
**10**	1.8	PB not present
**Mean ± SD**	2.02 ± 0.71	2.80 ± 1.01

*All branches lay posterior to medial border of tibia at level of tibial tuberosity
