# Supplementary material for: Distribution, course, and spatial relationships of the saphenous nerve: A 3D neuroanatomical map for nerve stimulation
Source: PLoS One. 2024 Feb 8;19(2):e0297680. doi: 10.1371/journal.pone.0297680 (PMC10852217; doi:10.1371/journal.pone.0297680)
Supplement: S3 Table — (PDF) [file pone.0297680.s003.pdf]

S3 Table. Distance of anterior branch (AB) from medial border of tibia at level of mid-point of leg by specimen.

Specimen	Distance: AB (cm)
**1**	0.71*
**2**	1.23
**3**	0.37
**4**	1.15
**5**	2.18
**6**	0.61
**7**	1.36
**8**	1.05
**9**	0.67
**10**	1.19
**Mean ± SD**	1.09 ± 0.53

*AB lay anterior to medial border of tibia at level of mid-point of leg; AB in specimens 2-10 lay posterior to the landmark
