# Supplementary material for: Distribution, course, and spatial relationships of the saphenous nerve: A 3D neuroanatomical map for nerve stimulation
Source: PLoS One. 2024 Feb 8;19(2):e0297680. doi: 10.1371/journal.pone.0297680 (PMC10852217; doi:10.1371/journal.pone.0297680)
Supplement: S4 Table — (PDF) [file pone.0297680.s004.pdf]

S4 Table. Distance of anterior branch (AB) from mid-point of anterior border of medial malleolus by specimen.

Specimen	Distance: AB (cm)*	
	Anterior	Posterior
**1**	0.8	-
**2**	-	1.5
**3**	-	1.6
**4**	-	2.1
**5**	1.4	-
**6**	-	0.9
**7**	1.1	-
**8**	0.9	-
**9**	-	1.6
**10**	0.5	-
**Mean ± SD**	0.94 ± 0.34	1.54 ± 0.43

*Distance between AB and mid-point of anterior border of medial malleolus
